# Phase 1 dose-escalation trial evaluating a group 2 influenza hemagglutinin stabilized stem nanoparticle vaccine

**DOI:** 10.1038/s41541-024-00959-0

**Published:** 2024-09-17

**Authors:** Joseph P. Casazza, Amelia R. Hofstetter, Pamela J. M. Costner, LaSonji A. Holman, Cynthia S. Hendel, Alicia T. Widge, Richard L. Wu, William R. Whalen, Jennifer Cunningham, Anita Arthur, Xiaolin Wang, Abidemi Ola, Jamie Saunders, Floreliz Mendoza, Laura Novik, Maria C. Burgos Florez, Ana M. Ortega-Villa, Preeti J. Apte, Larisa Strom, Lu Wang, Marjaan Imam, Manjula Basappa, Mursal Naisan, Mike Castro, Jessica F. Trost, Sandeep R. Narpala, Hillary A. Vanderven, Galina V. Yamshchikov, Nina M. Berkowitz, Ingelise J. Gordon, Sarah H. Plummer, Diane L. Wycuff, Sandra Vazquez, Rebecca A. Gillespie, Adrian Creanga, William C. Adams, Kevin Carlton, Jason G. Gall, Adrian B. McDermott, Leonid A. Serebryannyy, Katherine V. Houser, Richard A. Koup, Barney S. Graham, Julie E. Ledgerwood, John R. Mascola, Theodore C. Pierson, Sarah F. Andrews, Masaru Kanekiyo, Lesia K. Dropulic, The VRC 323 study team, Akuah Abrah, Seemal F. Awan, Obrimpong Amoa-Awua, Martin Apgar, Allison Beck, Katherine Brooks, Tommy Bruington, Eugenia Burch, Chris Case, Emily E. Coates, Erykah Coe, Aba M. Eshun, Martin R. Gaudinski, Renunda Dyer, Lam Le, Brenda Larkin, John Misasi, Patricia Morgan, Allen Mueller, Thuy Nguyen, Mark O’Callahan, Amine Ourahmane, Karen M. Parker, Iris Pittman, Matthew Reiber, LaShawn Requilman, Geoffrey Shimberg, Rosa Silva, Judy Stein, Shinyi Telscher, Jagada Thillainathan, Olga Vasilenko, Mingzhong Chen, Naga Chalamalasetty, Peifeng Chen, Bobby Boonyaratanakornkit, Robin Luedtke, Kristin Leach, Gabriel Arias, Michael Pratt, Krishana Gulla, Daniel B. Gowetski, Janel Holland-Linn, Leigh Anne Stephens, Paula Lei, Jessica Bahorich, Jonathan Cooper, Yanhong Yang, Eric Wang, William Shadrick, Lisa Kueltzo, Sashikanth Banappagari, Gabriela Albright, Gelu Dobrescu, Sean Nugent, Gabriel Moxey, Rajoshi Chaudhuri

**Affiliations:** 1 Vaccine Research Center, National Institute of Allergy and Infectious Disease, National Institutes of Health, Bethesda, MD 20892 USA; 2 U.S. Public Health Service Commissioned Corps, Rockville, MD 20852 USA; 3 Biostatistics Research Branch, Division of Clinical Research, National Institutes of Health, Bethesda, MD 20892 USA; 4 Australian Institute of Tropical Health and Medicine, James Cook University, Douglas, QLD 4811 Australia

**Keywords:** Translational research, Protein vaccines, Influenza virus

## Abstract

The relative conservation of the influenza hemagglutinin (HA) stem compared to that of the immunodominant HA head makes the HA stem an attractive target for broadly protective influenza vaccines. Here we report the first-in-human, dose-escalation, open-label trial (NCT04579250) evaluating an unadjuvanted group 2 stabilized stem ferritin nanoparticle vaccine based on the H10 A/Jiangxi-Donghu/346/2013 influenza HA, H10ssF, in healthy adults. Participants received a single 20 mcg dose (*n* = 3) or two 60 mcg doses 16 weeks apart (*n* = 22). Vaccination with H10ssF was safe and well tolerated with only mild systemic and local reactogenicity reported. No serious adverse events occurred. Vaccination significantly increased homologous H10 HA stem binding and neutralizing antibodies at 2 weeks after both first and second vaccinations, and these responses remained above baseline at 40 weeks. Heterologous H3 and H7 binding antibodies also significantly increased after each vaccination and remained elevated throughout the study. These data indicate that the group 2 HA stem nanoparticle vaccine is safe and induces stem-directed binding and neutralizing antibodies.

## Introduction

Between 2010 and 2023, it is estimated that influenza infections caused between 9.3 to 41 million illnesses, 100,000 to 710,000 hospitalizations, and 4,900 to 51,000 deaths annually in the United States^1^. Since the licensure of the first egg-based influenza vaccine in 1945, the primary influenza protective intervention is the use of hemagglutinin (HA)-directed vaccines. Influenza A HAs are phylogenetically divided into 2 groups, group 1 and group 2. Each group contains multiple HA subtypes, of which only H1 (group 1) and H3 (group 2) subtypes are currently circulating in humans. The immunodominance of the HA head results in an immune response directed primarily against the antigenically variable HA head. These anti-HA head responses are highly potent, but highly strain-specific, contributing to the suboptimal vaccine efficacy of current commercial influenza vaccines, which can be as low as 10% and rarely exceeds 60%^2^. In addition, the licensed vaccines provide little protection against influenza viruses with HA subtypes of pandemic potential that are not currently circulating in the human population such as H2, H5, H9, H7, and H10^2^.

The immunologically subdominant HA stem is an attractive target for vaccine design for multiple reasons. The HA stem contains viral fusion machinery which plays an essential role during viral entry into the host cell^3,4^. The HA stem is also far more conserved than the HA head across influenza subtypes and more resistant to antibody escape mutations^5–7^. Multiple broadly neutralizing antibodies (bnAbs) that target the stem have been identified both after influenza infection and vaccination, and the presence of anti-stem antibodies independently correlates with protection in humans^8–13^.

Multiple strategies have been used to design immunogens capable of inducing HA stem-directed antibodies. Vaccine concepts include chimeric HAs combining HA heads of non-circulating subtypes with the stem of seasonal HA subtypes^14–16^, multivalent display of stem peptides on virus-like particles^17^, and hyperglycosylation of the HA head region to dampen immune responses to it^18^. Another strategy to subvert the immunodominance of the HA head region is to design “headless” stem antigens^19–24^. Through structure-based design we and others have produced stabilized stem trimers that are both structurally and antigenically intact^19–24^. These stabilized stem trimers can be displayed on *Helicobacter (H.) pylori* ferritin nanoparticles, further enhancing their immunogenicity^22,24–26^. In a phase I vaccine clinical trial, the H1 stabilized stem ferritin nanoparticle (H1ssF) vaccine was safe, well tolerated, and induced bnAbs specific for group 1 subtypes^26^. However, H1ssF did not induce group 2 cross-reactive antibodies, demonstrating the need for immunogens that can generate protective antibodies against group 2 subtypes. Pre-clinical data indicated that a nanoparticle displaying the stabilized stem of H10 (H10ssF) provided superior protection to mice following group 2 influenza viral challenge compared to either H3 or H7 stabilized stem contructs^25^. Here, we report the results of the first-in-human vaccine trial in which volunteers were vaccinated with a group 2 H10ssF nanoparticle, derived from an H10N8 influenza virus (A/Jiangxi-Donghu/346/2013). The primary objective of the trial was to evaluate the safety and tolerability of the H10ssF vaccine. The secondary objective was to evaluate H10ssF-induced antibody responses.

## Results

### Trial design and participants

**Fig. 1:**
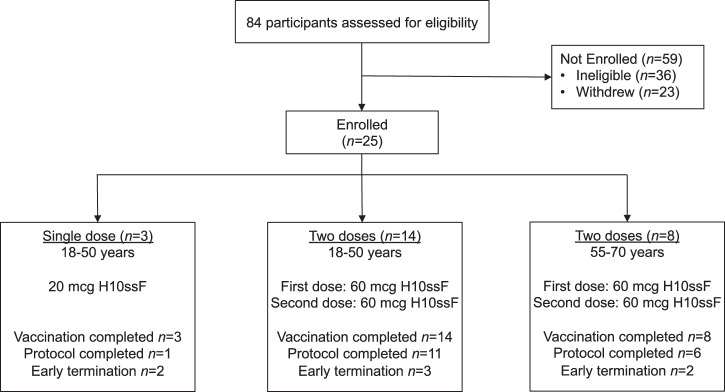
Study CONSORT Diagram. Twenty-five healthy adults aged 18–70 years old enrolled between 8 October 2020 and 22 April 2021 to receive 20 mcg H10ssF once (*n* = 3) or 60 mcg H10ssF twice (*n* = 22) with an interval of 16 weeks between vaccines. A total of 7 early terminations resulted from participant relocation (*n* = 4), early withdrawal due to time commitment (*n* = 1), or loss of participant to follow-up (*n* = 2). All participants were monitored for safety and immunogenicity until study completion or termination.

Between 8 October 2020 and 22 April 2021, we enrolled 25 healthy adults to receive the H10ssF vaccine (Fig. 1, Table 1). Participants received either a single 20 mcg dose, or two 60 mcg doses 16 weeks apart. All participants were followed for 40 weeks. Trial accrual was closed prior to the onset of the 2021 influenza season over concern of a prolonged enrollment period impacting participant baseline immunity. This resulted in incomplete enrollment in the single 20 mcg dose group and the 55–70 years cohort of the two 60 mcg dose groups. The participants had a mean age of 40 years (range 22–66) and were 60% male (*n* = 15). Most participants (80%) had received at least three seasonal influenza vaccines in the five years preceding the trial (Supplementary Table 1). The first three participants enrolled (18–50 years of age) each received a single 20 mcg dose of H10ssF. One participant completed all study visits; one was lost to follow-up after the week 12 visit; the third withdrew due to a time commitment after week 28. The trial escalated to a 60 mcg dose after the protocol safety review team reviewed the outcomes in the 20 mcg participants and assessed the vaccine to be safe for further investigation. Twenty-two participants each received two doses of 60 mcg H10ssF with a 16 week interval. Of these 22 participants, 17 (77%) completed all study visits. No participants withdrew from the trial due to safety or tolerability concerns. Five (23%) participants were lost to follow-up or withdrew between weeks 28 and 40 due to relocations or concerns over the time commitment required for participation (Fig. 1). The last study visit occurred on 27 January 2022.

**Table 1:** Participant demographics

	20 mcg H10ssF	60 mcg H10ssF			Total
	18–50 years (*n* = 3)	18–50 years (*n* = 14)	55–70 years (*n* = 8)	All 60 mcg (*n* = 22)	Overall (*n* = 25)
Sex – no. (%)					
Male	2 (66.7)	8 (57.1)	5 (62.5)	13 (59.1)	15 (60.0)
Female	1 (33.3)	6 (42.9)	3 (37.5)	9 (40.9)	10 (40.0)
Age – mean yr [range]	34 [29, 43]	30 [22,47]	61 [56, 66]	41 [22,66]	40 [22,66]
Race – no. (%)^a^					
Asian	0 (0.0)	4 (28.6)	1 (12.5)	5 (22.7)	5 (20.0)
Black or African American	1 (33.3)	3 (21.4)	0 (0.0)	3 (13.6)	4 (16.0)
White	2 (66.7)	6 (42.9)	6 (75.0)	12 (54.5)	14 (56.0)
Multiracial	0 (0.0)	1 (7.1)	1 (12.5)	2 (9.1)	2 (8.0)
Hispanic or Latino ethnic group – no. (%)^a^					
Non-Hispanic/Latino	3 (100.0)	10 (71.4)	6 (75.0)	16 (72.7)	19 (76.0)
Hispanic/Latino	0 (0.0)	4 (28.6)	2 (25.0)	6 (27.3)	6 (24.0)
Body-Mass Index – mean (S.D.)^b^	26.1 (2.9)	26.2 (4.0)	28.7 (3.4)	27.1 (3.8)	27.0 (3.7)
Educational Level – no. (%)					
College/University	0 (0.0)	8 (57.1)	4 (50.0)	12 (54.5)	12 (48.0)
Advanced degree	3 (100.0)	6 (42.9)	4 (50.0)	10 (45.5)	13 (52.0)

^a^Race and ethnic group were self-reported by the participants.

^b^Body-mass index is weight in kilograms divided by the square of height in meters. This calculation was performed based on weight and height measured at the time of enrollment.

### H10ssF vaccination was safe and well tolerated

Administration of the H10ssF vaccine in this trial revealed a favorable safety profile. Five asymptomatic adverse events (AEs) attributed to vaccination occurred (Supplementary Table 2). These included single cases of mild leukopenia, mild lymphopenia, and mild and moderate neutropenia, occurring 4 weeks after the first dose of vaccine. Additionally, a single mild AE of increased aspartate aminotransferase was detected 4 weeks after the second vaccination. Although the resolution of neutropenia in a 20 mcg dose recipient who was lost to follow-up could not be confirmed, all other vaccine-attributed AEs self-resolved by the following study visit. One influenza-like illness was reported in a 60 mcg dose recipient six days after their week 17 visit, but a nasal swab from the participant was PCR-negative for influenza virus.

**Fig. 2:**
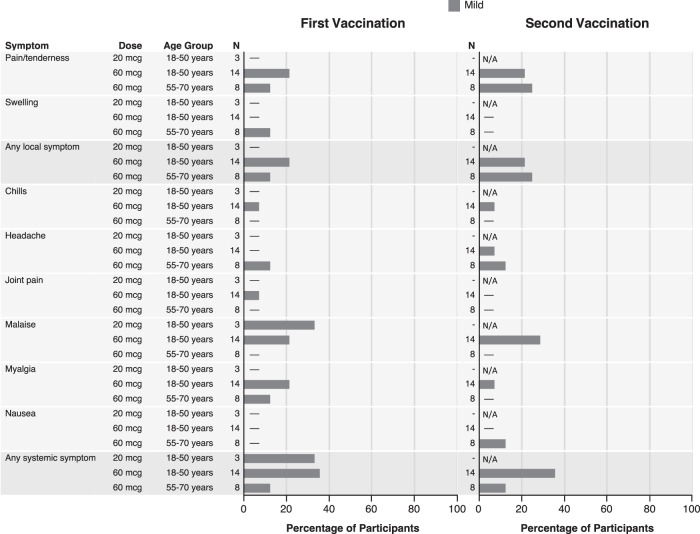
The H10ssF vaccine elicited mild reactogenicity. Percentage of participants (*x*-axis) reporting solicited reactogenicity symptoms (*y*-axis) in the seven days following each vaccination. For symptoms persisting more than 1 day, a single count per person at the maximum severity of the symptom was plotted. No injection site redness, pruritus, bruising, or fevers were reported. N/A indicates that no second vaccination was administered to the dose group.

The H10ssF vaccine was well tolerated in this trial (Fig. 2). The only solicited reactogenicity reported in the 20 mcg dose group was a single episode of mild malaise. Half (*n* = 11) of the 60 mcg dose recipients reported no reactogenicity after each vaccination and the remaining participants experienced only mild symptoms. The most frequently reported local reactogenicity symptom in the 60 mcg recipients was mild pain/tenderness at the injection site, reported by 8/22 (36%). The most common systemic symptom in the 60 mcg dose recipients was mild malaise (5/22, 23%), followed by myalgia (4/22, 18%). The incidence and severity of reported reactogenicity were similar after both the first and second vaccinations.

To explore the possibility that the *H. pylori* ferritin nanoparticle elicits a cross-reactive immune response against human ferritin, we measured antibody responses against *H. pylori* and human ferritin antigens following vaccination in an exploratory evaluation (Supplementary Fig. 1). The H10ssF vaccine elicited binding antibodies specific for the *H. pylori* ferritin component of the nanoparticle (week 20 compared to baseline, *p* < 0.0001). However, as observed previously with this vaccine platform^26,27^, binding antibodies specific for the antigenically distinct human ferritin proteins did not significantly increase over baseline levels.

### H10ssF elicited antibodies against autologous H10 HA

We next assessed the immunogenicity of the H10ssF by measuring the antibody responses to HA. Since the purpose of the 20 mcg dose group was to establish a safety profile for the H10ssF vaccine, the immunogenicity results for this group are separated from the 60 mcg groups and reported in the supplement (Supplementary Fig. 2).

**Fig. 3:**
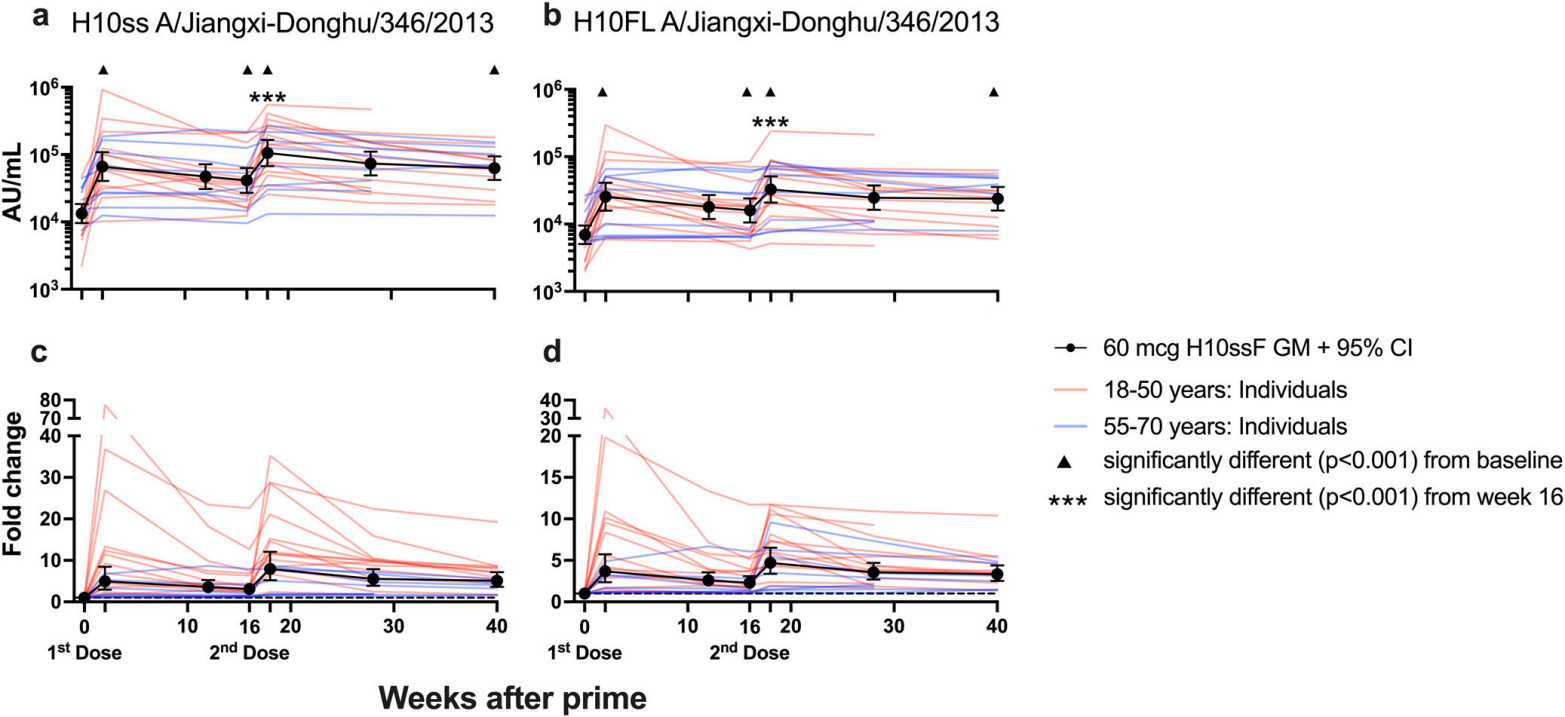
H10ssF vaccination elicited antibodies against homologous H10 HA antigens. Binding antibodies were assayed using H10 stabilized stem (H10ss, **a**, **c**) or full-length (H10FL, **b**, **d**) antigens by ECLIA. The AU/mL GM of the 60 mcg recipients’ binding antibodies (**a**, **b**) or fold change compared to baseline (**c**, **d**) are shown in black with 95% CI. Individual participants’ AU/mL are shown in red or blue lines based on their age group. In (**c**) and (**d**), the dashed line represents no fold change from baseline. Symbols in (**a**) and (**b**) indicate where the AU/mL GM values at week 2, 16, 18, or 40 after vaccination were significantly different (*p* < 0.001) from the AU/mL GM at day 0 or week 16.

Vaccine-induced homologous H10-binding serum antibody responses were assessed by electrochemiluminescence immunoassay (ECLIA, Fig. 3), with binding antibodies quantified in arbitrary units per milliliter (AU/mL, see Methods for details). Antibodies specific to H10 stabilized stem (H10ss) and H10 full-length (H10FL) were significantly (*p* < 0.001) increased from baseline at all timepoints following H10ssF vaccination (Fig. 3a, b). By week 2, binding antibodies for the H10ss antigen increased 5.0-fold (Fig. 3c). Similarly, H10FL binding responses increased 3.7-fold (Fig. 3d). The second H10ssF dose significantly (*p* < 0.001) increased the week 18 geometric means (GMs) of H10ss- and H10FL-binding antibodies from baseline (7.9-fold and 4.7-fold, respectively) and from the week 16 values (2.6-fold and 2.0-fold, respectively). These responses remained significantly elevated above baseline through 40 weeks (*p* < 0.001). In summary, the H10ssF vaccine elicited H10-specific binding antibodies following both the first and second vaccine administrations that remained elevated through the end of the study.

**Fig. 4:**
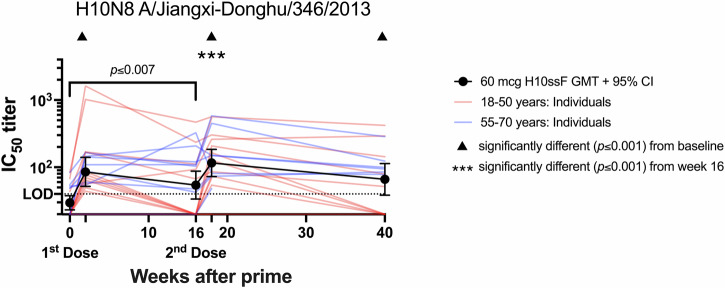
H10ssF vaccination elicited neutralizing antibodies against H10N8 A/Jiangxi-Donghu/346/2013. The geometric mean IC_50_ neutralizing antibody titers (GMT) and 95% CI of all 60 mcg H10ssF recipients are shown in black, with individual participants’ values in red or blue based on their age group. The dotted line indicates the assay LOD. Brackets indicate two time points that are significantly different from each other with the *p* value noted above the line. When the significance between a timepoint and baseline is *p* ≤ 0.001, this is noted by a symbol above the later timepoint. When LOD imputation with zero produced a different *p* value than imputation with half LOD, results are reported as *p* ≤ *x*, where *x* is the least significant *p* value resulting from the two test methods.

Homologous H10N8 neutralizing antibody titers were also significantly increased (*p* ≤ 0.007) at all timepoints after vaccination (Fig. 4), as evaluated by a reporter microneutralization assay^28^. More than half (14/22, 64%) of participants had H10N8 neutralizing antibody responses below the assay limit of detection (LOD, IC_50_ of 40) at baseline; however, 17 of 22 (77%) 60 mcg dose participants responded to the H10ssF vaccine with detectable neutralization titers by week 2. The geometric mean titer (GMT) for the 60 mcg dose group at week 2 was IC_50_ of 85 (95% CI: 52–140). The second dose further increased the H10N8 neutralizing GMTs to IC_50_ of 116 at week 18, a significant increase from both baseline (*p* < 0.001) and the week 16 GMT IC_50_ of 54 (95% CI: 34–87), (*p* ≤ 0.001). These responses remained significantly elevated over baseline by 40 weeks after vaccination (*p* = 0.001). Similar kinetics were observed when the data were assessed in IC_80_ titers, including significant increases in H10-neutralizing antibodies over baseline at weeks 2, 16, 18, and 40, as well as a significant increase at week 18 over week 16 (Supplementary Fig. 3). However, the group GM IC_80_ titer was below the assay LOD at week 2. Together the data demonstrate that each 60 mcg administration of the H10ssF vaccine increased the magnitude of binding and neutralizing antibody responses to H10 HA, which remained significantly elevated throughout the duration of the study.

Animal studies have fostered an increasing appreciation of the protective role of Fc-mediated effector functions in influenza infection^29^. Studies in mice have shown a role of Fc-mediated effector functions for both head- and stem-directed antibodies in protective responses to influenza virus^30,31^. To expand upon the microneutralization results and to confirm the presence of functional H10-specific antibodies, participant serum was assessed in a dimeric FcγRIIIa assay (Supplementary Fig. 4). FcγRIIIa is the only activating Fc receptor found on NK cells and is therefore critical for antibody-dependent cellular cytotoxicity (ADCC)^32,33^. The dimeric FcγRIIIa assay was previously shown to correlate with ADCC activity^34^. The geometric mean dimeric FcγRIIIa binding response was significantly increased at all timepoints evaluated following vaccination (Supplementary Fig. 4a), peaking 1.7-fold over baseline levels at week 18 (Supplementary Fig. 4b). This suggests that the polyclonal H10ssF-elicited serum may include antibodies with FcγRIIIa-mediated functionality in addition to direct virus neutralization.

### H10ssF elicited cross-reactive antibody responses against group 2 influenza HAs

**Fig. 5:**
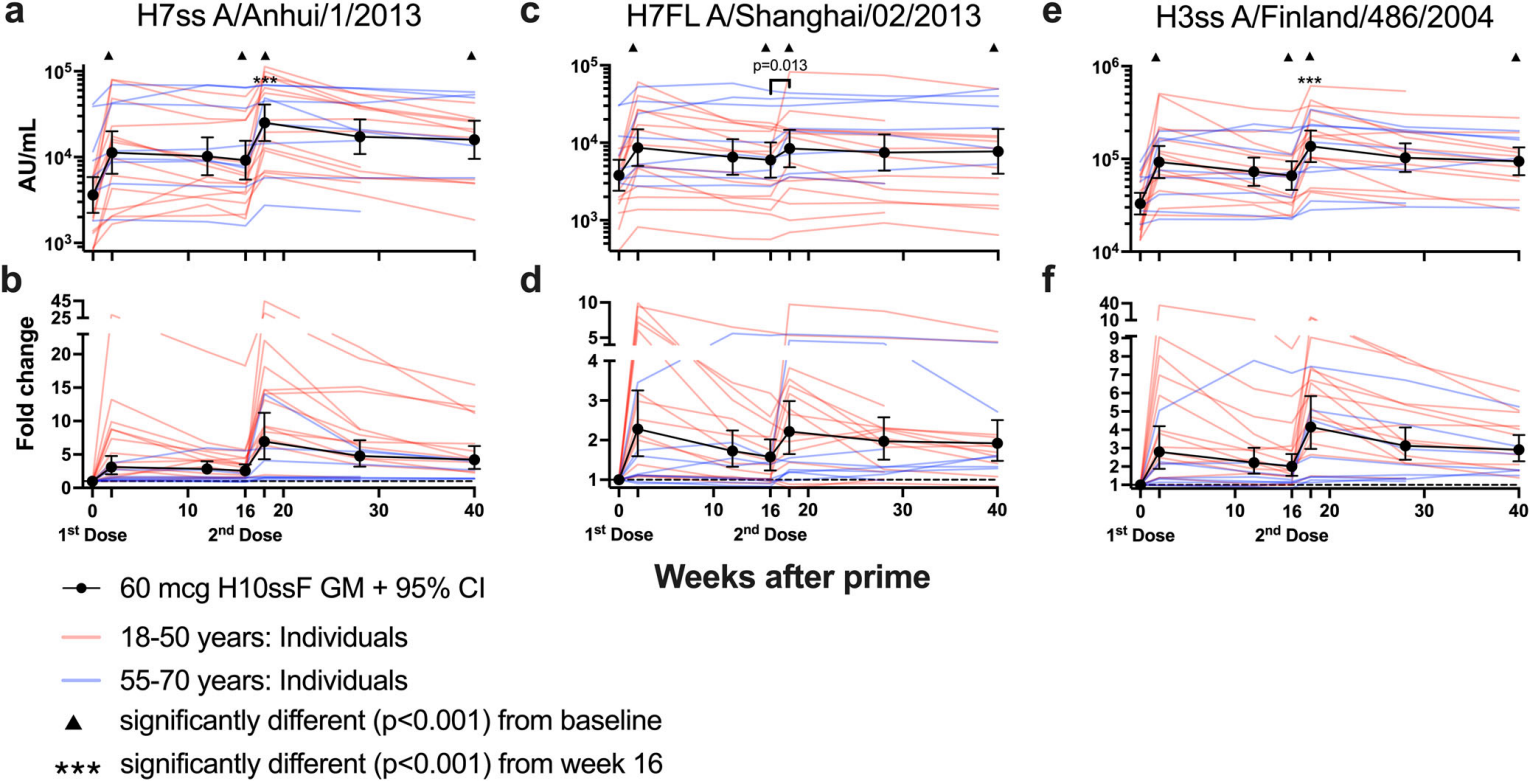
H10ssF vaccination elicited binding antibodies against heterologous group 2 influenza HAs. Binding antibodies were assayed using the H7 stabilized stem (H7ss, **a**, **b**) or full-length (H7FL, **c**, **d**) antigens, or the H3ss antigen (**e**, **f**) by ECLIA. The GM of the 60 mcg recipients’ binding antibodies in AU/mL (**a**, **c**, **e**) or fold change (**b**, **d**, **f**) are shown with 95% CI in black, with individual participants’ AU/mL in red or blue based on their age group. In (**b**), (**d**), (**f**), the dashed line represents no change over baseline. Brackets indicate two time points that are significantly different from each other, with the *p* value noted above the line. When the significance between a timepoint and baseline is *p* < 0.001, this is noted by a symbol above the later timepoint.

Given the goal of increasing broad stem-directed antibody titers with this vaccine platform, the antibody responses against additional influenza HA antigens were also investigated. H10ssF vaccination significantly increased heterologous binding antibody AU/mL GMs for other group 2 influenza antigens, including H7ss, H7FL, and H3ss (Fig. 5) at all timepoints (*p* < 0.001). The binding antibody AU/mL GMs for the H7ss, H7FL, and H3ss antigens at week 2 were 3.1-, 2.3-, and 2.8-fold over baseline, respectively. The second dose significantly increased the antibody GMs at week 18 compared to the week 16 samples (*p* < 0.001 for H7ss and H3ss antigens, *p* = 0.013 for H7FL). Additionally at week 18, the binding antibody GMs for H7ss, H7FL, and H3ss antigens were 6.9-, 2.2-, and 4.2-fold over baseline, respectively. However, there was little to no increase in neutralizing antibodies to H3N2 viruses (Supplementary Fig. 5) and H7N9-specific neutralizing antibodies were not observed in any participant following vaccination. No significant increases in binding antibody GMs were observed for group 1 influenza antigens at any time after infection (Supplementary Fig. 6), nor were neutralizing GMTs increased for H1N1 A/Michigan/45/2015 (Supplementary Fig. 7). Although no H7N9-specific neutralizing antibodies were observed, the dimeric FcγRIIIa assay detected H7FL-specific antibodies in some participants (Supplementary Fig. 8). Together these data demonstrate that the H10ssF vaccine potently elicits a broadly cross-reactive group 2 influenza HA antibody response with limited neutralization breadth.

### H10ssF elicited equivalent immune responses in younger and older adults

Recent data suggests that an individual’s first influenza exposure can skew subsequent responses towards stronger influenza HA group 1 or group 2 immunity^35^. We hypothesized that participants born after the group 2 H3N2 subtype emerged as a pandemic strain in 1968 might have improved immune responses to H10ssF when compared to participants initially imprinted only with group 1 influenza viruses. Therefore, participants were recruited from birth years prior to 1965, when only group 1 (H1N1 and H2N2) influenza subtypes were circulating (ages 55–70 at the time of the trial) or after 1970, following the 1968 group 2 H3N2 influenza pandemic (ages 18–50 at the time of the trial). However, few significant differences between age cohorts were observed in a protocol-specified subgroup comparison. Older individuals had higher baseline binding antibody GMs to the group 2 H7ss, H7FL, and group 1 H5ss antigens (Supplementary Fig. 9). For the H5ss antigen, this baseline difference (*p* = 0.026) was maintained, with the 55–70-year-old participants displaying a significantly higher GM at weeks 16 and 40 after the first vaccination (*p* = 0.049 and *p* = 0.043, respectively) as compared to 18–50 years old participants, underscoring our observation that H10ssF vaccination did not affect immune responses to this antigen. In contrast, the baseline differences for the H7 antigens (*p* = 0.013 for H7ss and *p* = 0.021 for H7FL) were largely eliminated following vaccination, with the only significant difference detected after vaccination being greater H7FL-specific antibody GMs among the older cohort at week 40 (*p* = 0.023). No other significant differences between the age groups were observed for any binding or neutralizing antibody responses in the trial. Overall, no detectable impact of age-based immune imprinting was observed on vaccine-elicitation of group 2 stem antibodies after H10ssF vaccination.

## Discussion

Here we report the results of the first-in-human phase I clinical trial evaluating a group 2 influenza stabilized stem nanoparticle vaccine. This immunogen, a ferritin nanoparticle displaying eight H10 HA stem trimers from A/Jiangxi-Donghu/346/2013 (H10ssF), had a favorable safety profile and was immunogenic. Solicited local and systemic reactogenicity was mild in all trial participants. Administration of a 60 mcg dose of H10ssF significantly and durably increased binding antibody titers above baseline levels for group 2 antigens (including H10ss, H10FL, H7ss, H7FL, and H3ss) after both the first and the second vaccinations through 40 weeks after the first vaccination. Vaccination also induced anti-stem antibody-mediated modest increases in homologous H10N8 neutralizing antibody titers. Vaccination with H10ssF did not induce antibody binding responses to the group 1 antigens studied (H1ss or H5ss). In this small study, we saw no evidence of age-based antigen imprinting in the response to vaccination with H10ssF. Overall, the H10ssF vaccine was safe and well tolerated in this trial population and elicited antibody responses against the group 2 influenza antigens evaluated.

The data show that the H10ssF vaccine induced stem-directed antibodies in serum as measured by both binding and functional antibody assays. The ECLIA demonstrated broad group 2 influenza responses to not only the homologous H10ss antigen, but also to heterologous group 2 influenza stabilized HA stems derived from H7N9 A/Anhui/1/2013 and H3N2 A/Finland/486/2004. At least a four-fold increase in binding antibodies was detected for all three group 2 influenza stem antigens. Although the removal of the HA head and introduction of stabilizing changes in the stem amino acid sequence could result in immunologically novel epitopes on the H10ssF vaccine, the vaccine elicited binding antibodies against full-length H7 and H10 HA antigens, demonstrating that H10ssF does induce antibodies against native group 2 stem epitopes. Additionally, vaccination with H10ssF elicited an H10N8 neutralizing antibody response with similar kinetics to the binding antibody responses.

In our recent phase 1 trial evaluating the group 1 H1ssF vaccine^26^, H1ssF induced broadly binding antibodies to group 1 H1, H2, and H5 stem antigens and increased neutralizing titers against H1N1, H2N2, and H5N1 reporter viruses. In contrast, the immunogen used in this trial induced limited neutralizing responses against group 2 H3N2, H7N9, and H10N8 reporter viruses. Several factors may contribute to the limited generation of broadly neutralizing antibodies by H10ssF compared to the group 1 H1ssF vaccine. It is generally accepted that initial adult vaccine responses are driven by the activation of pre-existing memory B and plasma cells^36^. It is possible that differences in prior human exposure to the vaccine antigens used in these two trials may have contributed to these disparate results. Humans have been repeatedly exposed to the H1ssF vaccine-matched seasonal H1 subtype, whereas the H10ssF vaccine relies on a preexisting memory repertoire to the vaccine-mismatched seasonal H3 subtype, as only nine human H10 influenza infections have been reported worldwide^37^. Consequently, unlike the H1ssF vaccination, there is no H10 primary response to boost. More importantly, most isolated and characterized broadly neutralizing stem antibodies are specific to group 1 HAs^38,39^. The lower threshold development path for group 1 HA broadly neutralizing stem antibodies such as those of the VH1-69 antibody lineage may also contribute to their more efficient induction. Fifty to 60% of the identified group 1 stem-directed neutralizing antibodies^36^ are derived from the VH1-69 gene, in which the germline-encoded CDR H2 contacts group 1 HA stems with only the antibody heavy chain^40^ and requires only modest somatic hypermutation for high affinity binding and neutralization^41^. The generation of group 2 HA stem-directed bnAbs appears to be more diverse and restricted^41^. Therefore, a group 2 stabilized stem vaccination may have fewer pre-existing cross-reactive group 2 responses to boost than a group 1 stabilized stem vaccine. Although difficult to induce, cross-reactive group 2 antibodies have been identified after influenza infection and vaccination. The use of adjuvants or the use of an HA stem from a different group 2 subtype may improve these neutralizing antibody responses.

Some stem-directed reactive antibodies require IgG Fc receptor (FcγR) engagement for optimal protection in vivo^42^. Indeed, prior small animal studies show discordance between in vitro neutralization activity and in vivo protective efficacy that may be explained by Fc-mediated functions^30,31^. Accordingly, a dimeric FcγRIIIa assay, which is a correlative readout of ADCC activity^34^, identified increases in FcγRIIIa-cross-linking functionality following H10ssF vaccination. Additional vaccine-induced Fc-mediated effector functions, including complement C1q deposition (shown to enhance HA stem-directed neutralizing activity)^43^, may also have been missed by our readouts.

The small number of participants, unblinded dosing, and lack of multiple comparison adjustments, while typical for a phase 1 clinical trial, do limit the strength of our conclusions. Importantly, this trial was powered for the primary safety and secondary immunogenicity endpoints of the trial, not for efficacy. The COVID-19 pandemic suppressed enrollments, and the trial was closed to accrual before a new influenza season could confound the baseline immunity of the participant pool. Therefore, the group sizes were smaller than intended. Nonetheless, this trial revealed significant vaccine-induced elicitation of stem-directed antibodies to group 2 HA subtypes in the participants following vaccination with H10ssF.

The limited efficacy and required annual reformulations of the currently licensed influenza vaccines make the improvement of influenza vaccines a priority. Although a truly universal influenza vaccine is beyond the scope of current approved vaccine technology, ongoing efforts from our group and others aim to improve upon the available influenza vaccine platforms. Self-assembling stabilized stem nanoparticles offer the advantage of enabling the boosting of heterosubtypic antibodies and are amenable to mRNA vaccine formulation. There is an ongoing trial evaluating the group 1 (H1ssF) vaccine in a mRNA vaccine format (NCT05755620). This trial and our previous study using a group 1 stabilized stem ferritin nanoparticle^26^ serve as proof of principle that group 1 and group 2 stabilized stem nanoparticle vaccines are safe and well tolerated and capable of inducing both stem-directed binding and neutralizing antibodies. A cocktail of group 1 and group 2 stabilized stem nanoparticles elicits both group 1 and group 2 HA as well as cross-group stem-directed antibody responses in preclinical animal models^25^, and is being evaluated in an ongoing trial (NCT05155319). Our pre-clinical and clinical trial results demonstrate the usefulness of a headless group 2 stabilized influenza HA stem construct in expanding subdominant and cross-reactive stem-specific immune responses.

## Methods

### Experimental design

This phase 1, open-label, dose escalation clinical trial (ClinicalTrials.gov, NCT04579250) was designed to evaluate the safety, tolerability, and immunogenicity of H10ssF in healthy adults. The primary objectives were evaluation of safety and tolerability of the vaccine after a single 20 mcg dose or two 60 mcg doses administered 16 weeks apart. The secondary objectives were evaluation of the antibody responses at two weeks after each vaccination with 20 mcg or 60 mcg H10ssF. The doses were chosen based on prior clinical experience with the ferritin nanoparticle vaccine platform^26^. Group sizes were selected to ensure the ability to detect severe adverse events and to allow for meaningful immunogenicity studies. For the 20 mcg dose participants (*n* = 5), there was a 90% chance to observe at least one SAE if the true rate was at least 0.369 and a more than 90% chance to observe no SAE if the true rate was less than 0.021. For the 60 mcg dose participants, within each age cohort (*n* = 15), there was a greater than 90% chance to observe at least one SAE if the true rate was at least 0.145 and a more than 90% chance to observe no SAE if the true rate was no more than 0.0069. To assess the primary safety and tolerability endpoints, participants recorded solicited local and systemic reactogenicity for seven days following vaccination, and clinical and laboratory assessments were conducted at protocol-specified visits for 40 weeks. Unsolicited AEs were recorded for 28 days after each vaccination, graded according to a modified FDA Toxicity Grading Scale for Healthy Adults and Adolescent Volunteers Enrolled in Preventative Vaccine Clinical Trials (see Protocol). Influenza-like illness (ILI), serious adverse events, and new chronic medical conditions were recorded for the duration of the clinical trial, which lasted 40 weeks after the first vaccination. Nasopharyngeal swabs were collected for ILIs for laboratory confirmation of influenza infection by PCR. Secondary objectives included the evaluation of H10 stem-specific antibody responses at two weeks after each vaccination.

The H10ssF (VRC-FLUNPF0103-00-VP) vaccine is a ferritin nanoparticle displaying eight stabilized-stem HA trimers from A/Jiangxi-Donghu/346/2013 (H10N8, equivalent to A/Jiangxi-Donghu/IPB13/2013) influenza. *H. pylori* non-heme ferritin spontaneously assembles into stable nanoparticles, allowing influenza HA protomers expressed on each ferritin monomer to associate and properly fold into HA trimers^44^. The H10ss antigen was developed by replacing the H10 head with a glycine-rich linker, followed by a series of structure-based mutations to improve expression and immunogenicity^24,25^. The H10ssF vaccine was developed at the Vaccine Production Program Laboratory (VPPL) and produced at the VRC pilot plant (operated under contract by the Vaccine Clinical Materials Program, Leidos Biomedical Research, Frederick, MD) under Good Manufacturing Practices. The vaccine was vialed at 0.7 ± 0.1 mL in 3 mL glass vials at a concentration of 180 ± 36 mcg/mL. The formulation buffer consisted of 20 mM sodium phosphate, pH 7.2, 100 mM sodium chloride, 5% w/v sucrose, and 0.01% w/v Pluronic F-68 (Poloxamer 188). The vaccine was stored frozen at −35 °C to −15 °C and thawed immediately prior to use. To administer the 20 mcg dose, the vaccine was diluted in sterile PBS, whereas no dilution was needed to administer the 60 mcg dose.

H10ssF was administered by needle and syringe into the deltoid muscle. Participants were observed for a minimum of 30 min following each injection, including recording vital signs and local reactogenicity. Safety was first established at 20 mcg. Once the protocol safety review team (PSRT) found no safety concerns by two weeks after the first three participants received 20 mcg, a dose-escalation ensued with enrollment of 18–50-year olds to receive the 60 mcg dose. An additional safety review per protocol occurred following two weeks of safety observations for the first three recipients of 60 mcg H10ssF before enrollment was opened for administration of second doses of 60 mcg H10ssF and for enrollment of the older cohort of individuals, aged 55–70 years. Participants aged 51–54 at the start of the trial (birth years 1965 to 1970) were excluded to establish two distinct cohorts based on likely first influenza exposure.

The trial was conducted at the NIH Clinical Center by the VRC Clinical Trials Program, NIAID, NIH in Bethesda, MD. Participants were recruited from the greater Washington, DC area with NIH institutional review board (IRB)-approved written and electronic media, advertisement campaigns, and community outreach strategies. Inclusion criteria specified the selection of adults aged 18–70 (exclusive of individuals born between January 1, 1965, and December 31, 1970), in general good health, and who had received at least one licensed influenza vaccine from 2015 to the time of enrollment. Exclusion criteria included receipt of a previous investigational H10 influenza vaccine and receipt of a licensed influenza vaccine within 6 weeks prior to enrollment. Full exclusion and inclusion criteria are included in the study protocol (https://storage.googleapis.com/ctgov2-large-docs/50/NCT04579250/Prot_SAP_001.pdf). Written informed consent was obtained from all participants prior to enrollment. The study followed guidelines for conducting clinical human subjects research in accordance with 45 CFR Part 46 from the US Department of Health and Human Services, US Food and Drug Administration regulations for investigational products, and principles expressed in the Declaration of Helsinki. The clinical trial protocol was reviewed and approved by the NIH IRB.

### Immunological Methods

Participants’ serum samples were collected for immunogenicity analyses between baseline and 40 weeks after vaccination. HA binding antibodies were assayed using the Meso Scale Diagnostics (MSD) electrochemiluminescence (ECLIA) 10-Assay U-PLEX Development Pack (Cat# K15235N). Protein ligands were biotinylated at an AviTag site located proximal to the C-terminus from the trimer foldon, coupled to a unique streptavidin U-PLEX linker at 10 mcg/mL, and combined to form a multiplexed coating solution according to the manufacturer’s instruction. The stabilized stem proteins included: H1ss: A/Michigan/45/2015, H3ss: A/Finland/486/2004, H5ss: A/Indonesia/05/2005, H7ss: A/Anhui/1/2013, and H10ss: A/Jiangxi-Donghu/346/2013. The full-length proteins were H7FL: A/Shanghai/02/2013 and H10FL: A/Jiangxi-Donghu/346/2013. 96-well U-PLEX plates were coated for 1 h shaking at room temperature (RT). Following coating, plates were washed and incubated with serially diluted test samples and reference standard for 1 h shaking at RT. 315-53-1F12 (group 1 and 2 binding) antibody was used as a reference standard^28^. The binding of 50 ng/mL of 315-53-1F12 to H10ss was assigned a value of 100 arbitrary units per milliliter (AU/mL)^26^. AU/mL values for 315-53-1F12 binding to the heterologous HAs were assigned relative to H10ss binding. After sample incubation, plates were washed and 0.25 mcg/mL SULFO-TAG (MSD, cat #R91AO-1)-labeled anti-human IgG, IgM, and IgA (H + L) secondary antibody (ThermoFisher Cat#31128, RRID AB_228255, lot #0031128) was added for 1 h, shaking at RT. After the detection antibody incubation, plates were washed and a 1X MSD Read Buffer was applied, and plates were analyzed using the MSD Sector Imager S600. All samples were tested in duplicate. Samples with a replicate coefficient of variation > 30% were retested. Serial dilutions of samples within the dynamic range of the standard curve were interpolated to determine a sample’s AU/mL. Results were plotted and analyzed using Prism version 8 or newer (GraphPad, San Diego, CA).

Anti-ferritin binding antibodies were measured by MSD ECLIA. MSD 96-well bare plates (Cat# L15XA-3) were coated with respective ferritin proteins for 16–24 h at 4 °C. Ferritin antigens used were those previously described^26,27^. Following the overnight incubation, plates were washed and blocked in 5% bovine serum albumin for 1 h, shaking at RT. Following blocking, plates were washed and incubated with serially diluted test samples (1:100 followed by seven 3-fold serial dilutions) and control for 2 h shaking at RT. Plates were then washed and 5 mcg/mL SULFO-TAG (MSD, cat #R91AO-1)-labeled anti-human IgG, IgM, and IgA (H + L) secondary antibody (ThermoFisher Cat#31128, RRID AB_228255, lot #0031128) was added for 1 h, shaking at RT. After the detection antibody incubation, plates were washed, 1X MSD Read Buffer was applied, and plates were analyzed using the MSD Sector Imager S600. All samples were tested in duplicate. Serial dilutions of the sample were used to calculate and assign an area under the curve (AUC) value as the primary readout. Results were plotted and analyzed using Prism version 8 or newer (GraphPad, San Diego, CA). Samples with a replicate coefficient of variation >30% were retested.

Generation of the replication-restricted reporter (R3ΔPB1) virus subtypes H1N1, H3N2 and H10N8, as well as “rewired” replication-restricted reporter (R4ΔPB1) virus H7N9 is described elsewhere^28^. Briefly, to produce the R3ΔPB1 viruses the viral genomic RNA encoding functional PB1 was replaced with a gene encoding the fluorescent protein (TdKatushka2), whereas R4ΔPB1 H7N9 virus has the PB1 and H1 viral segments modified (“rewired”) to prevent HA reassortment: the HA coding region was inserted between PB1 genomic packaging signals and the fluorescent protein TdKatushka2 between the HA genomic packaging signals. The R3/R4ΔPB1 viruses were rescued by reverse genetics and propagated in the complementary cell line which expresses PB1 constitutively. Each virus stock was titrated by determining the fluorescent units per mL (FU/mL) prior to use in the experiments. For virus titration, serial dilutions of virus stock in OptiMEM were mixed with pre-washed MDCK-SIAT1-PB1 cells (8 × 10^5^ cells/ml) and incubated in a 384-well plate in quadruplicate (25 mcL/well). Plates were incubated for 18–26 h at 37 °C with 5% CO_2_ humidified atmosphere. After incubation, fluorescent cells were imaged and counted by using a Celigo Image Cytometer (Nexcelom) with a customized red filter for detecting TdKatushka2 fluorescence.

Neutralization activity of serum antibodies was determined using a reporter virus microneutralization assay as previously described^26,28^. Briefly, serial dilutions of Receptor Destroying Enzyme-treated serum were mixed with an equal volume of R3/R4ΔPB1 virus. The reporter viruses used were H10N8: A/Jiangxi-Donghu/346/2013, H7N9: A/Anhui/1/2013, H3N2v: A/Indiana/10/2011, H3N2: A/Singapore/INFIMH-16-0019/2016, and H1N1: A/Michigan/45/2015. After incubation, pre-washed MDCK-SIAT1-PB1 cells were added to the serum-virus mixtures and transferred to 384-well plates in quadruplicate (25 mcL/well). Plates were incubated and imaged as described above. The percent neutralization was calculated for each well by constraining the virus control (virus plus cells) as 0% neutralization and the cell-only control (no virus) as 100% neutralization. A 7-point neutralization curve was plotted against serum dilution for each sample, and a five-parameter logistic curve fit generated using LabKey was used to estimate the 50% (IC_50_, dilution^−1^) or 80% (IC_80_, dilution^−1^) inhibitory concentrations. Of note, serum dilutions take into account the sera concentrations after mixing with virus at 1:1 ratio. The limit of detection (LOD) of the assay is 40 dilution^−1^; one-half the LOD (20 dilution^−1^) was used to impute values below the LOD. For the calculation of GMTs, the results using the one-half LOD imputation were reported. Because most participants had an H10 neutralizing antibody titer below the LOD at baseline, statistical comparisons between time points for the H10 assay were subjected to a sensitivity analysis. Substitution of zero instead of half LOD did not change the conclusions of statistical testing. When different imputation methods changed the *p* value, the significance was reported as p ≤ *x*, where *x* is the least significant *p* value resulting from the two test methods.

The capacity of anti-HA antibodies in participant sera to bind human FcγRIIIa was measured using a recombinant soluble human FcγRIIIa (rsFcγRIIIa) dimer ELISA as previously described^45^. Briefly, 50 ng of recombinant H10 HA protein for the H10N8: A/Jiangxi-Donghu/346/2013 virus or recombinant H7 HA from the H7N9: A/Shanghai/02/2013 virus was coated in the wells of 96-well NUNC Maxisorp plates (Thermo Fisher Scientific, Waltham, MA, USA) overnight at 4 °C. The wells of the plates were blocked with 1% bovine serum albumin (BSA; Sigma-Aldrich, St. Louis, MO, USA) and 1 mM EDTA (Sigma-Aldrich, St. Louis, MO, USA) in phosphate-buffered saline for 1 h at 37 °C. Two-fold serial dilutions of participant sera, starting at a 1:10 dilution, were added to the wells of the plate and incubated for 1 h at 37 °C. Plates were washed then incubated with 50 mcL of 0.1 mcg/mL biotinylated human rsFcγRIIIa dimer for 1 h at 37 °C. After washing, plates were incubated with a 1:10,000 dilution of Pierce High Sensitivity HRP-Streptavidin (Thermo Fisher Scientific, Waltham, MA, USA) for 1 h at 37 °C then washed. The color was developed by adding 3,3,5,5-tetramethylbenzidine (TMB; Sigma-Aldrich, St. Louis, MO, USA) then stopped with 1 M hydrochloric acid (HCl), and absorbance read at 450 nm on a SPECTROstar Nano microplate reader (BMG Labtech).

### Statistical methods

Binding and neutralizing antibody titers were log_10_-transformed before analysis, after which data was assumed to be normally distributed. Binding antibody AU/mL GM or neutralizing antibody GMT and corresponding 95% CIs were calculated for each antigen at each time point. Comparisons of antibody AU/mL or neutralization titers between weeks were made using paired *t* tests. Fold changes and corresponding 95% CIs were calculated. Between group comparisons were performed pairwise at each available time point using two-sample *t* tests unless noted otherwise and are exploratory because of small group sample sizes. For the microneutralization assay, values at the LOD were imputed to half the LOD. GMT and corresponding 95% CIs were calculated for each antigen at each time point. To determine whether the H10ssF vaccine increases ferritin binding antibody responses, two-sided Wilcoxon Sign-Rank tests were performed stratified by group. For the FcγRIIIa analysis, absorbance readouts over the entire dilution series were used to calculate an area under the curve (AUC) for each sample. The AUC calculation was performed using the trapezoid method in R using the package DescTools^46^. AUCs were log_10_-transformed before analysis. AUC GMs and corresponding 95% CIs were calculated at each time point. Fold-changes between visits with corresponding 95% CIs were calculated, and comparisons between weeks were made using paired *t* tests. Between group comparisons were performed pairwise at each available time point using two-sample *t* tests. All analyses were two sided and results were deemed significant if the *p* value < 0.05. No multiple comparison adjustments were employed per protocol. R version 4.3.0 was used to analyze the microneutralization and ADCC data. R version 4.2.1 was used to analyze the anti-ferritin and ECLIA data.

## Supplementary information

Supplementary File

## Data Availability

All data associated with this study are present in the paper or the Supplementary Materials. Results generated in this study are available as de-identified data at ClinicalTrials.gov, NCT04579250. The study protocol, statistical analysis plan, and informed consent form are all available on ClinicalTrials.gov (https://www.clinicaltrials.gov/study/NCT04579250). Individual data that underlie the results reported in this article are available, after de-identification, in the Supplementary Materials. Additional data may be made available upon reasonable request to the corresponding author and jstein@mail.nih.gov for MTA requests, for investigators whose proposed use of the data has been approved by the NIH IRB. All unique materials used in this study are available from the authors upon reasonable request.
