# Supplementary material for: Phase 1 dose-escalation trial evaluating a group 2 influenza hemagglutinin stabilized stem nanoparticle vaccine
Source: NPJ Vaccines. 2024 Sep 17;9:171. doi: 10.1038/s41541-024-00959-0 (PMC11408684; doi:10.1038/s41541-024-00959-0)
Supplement: Supplementary file 1 — Supplementary File [file 41541_2024_959_MOESM1_ESM.pdf]

Supplementary Appendix

Table of Contents

TABLE OF CONTENTS 1

SUPPLEMENTARY TABLES 2

Supplementary Table 1: Influenza vaccination history 2
Supplementary Table 2: Adverse events related to H10ssF vaccination 3

SUPPLEMENTARY FIGURES 4

Supplementary Figure 1: H10ssF vaccination did not elicit antibody responses against human ferritin antigens. 4
Supplementary Figure 2: Binding and neutralizing antibody responses following a single 20 mcg dose of H10ssF. 5
Supplementary Figure 3: H10ssF vaccination elicited neutralizing antibodies against H10N8 A/Jiangxi-Donghu/346/2013 influenza virus. 6
Supplementary Figure 4: H10ssF vaccination elicited H10-specific antibodies with Fc-mediated activity. 7
Supplementary Figure 5: H3N2-neutralizing antibody response to H10ssF vaccination 8
Supplementary Figure 6: Significant increases in geometric mean binding antibody responses were not observed against group 1 influenza HAs following H10ssF vaccination. 9
Supplementary Figure 7: H10ssF vaccination did not elicit neutralizing antibodies against H1N1 A/Michigan/45/2015. 10
Supplementary Figure 8: H10ssF vaccination elicited H7 cross-reactive antibodies with Fc-mediated activity. 11
Supplementary Figure 9: Participant age had little to no impact on antibody responses for all antigens tested. 12

Supplementary Tables

Supplementary Table 1: Influenza vaccination history

	**20 mcg H10ssF 18-50 years (n=3)**	**60 mcg H10ssF 18-50 years (n=14)**	**60 mcg H10ssF 55-70 years (n=8)**	**Overall (n=25)**
Frequency of influenza vaccination in the previous 5 years – no. (%)				
1-2 times	0 (0.0%)	3 (21.4%)	2 (25.0%)	5 (20.0%)
3-5 times	2 (66.7%)	8 (57.1%)	5 (62.5%)	15 (60.0%)
>5 times	1 (33.3%)	3 (21.4%)	1 (12.5%)	5 (20.0%)
Most recent influenza vaccine – no. (%)				
2018/2019	0 (0.0%)	2 (14.3%)	0 (0.0%)	2 (8.0%)
2019/2020	1 (33.3%)	2 (14.3%)	0 (0.0%)	3 (12.0%)
2020/2021	2 (66.7%)	10 (71.4%)	8 (100.0%)	20 (80.0%)

No participants had influenza-like illness in the previous 12 months or swab-proven influenza in the past 5 years.

Supplementary Table 2: Adverse events related to H10ssF vaccination

Participant	Vaccination Group	Related Adverse Event	Severity	Days Post-Vaccination	Time from AE Onset to Resolution*
1	20 mcg	Neutropenia	Moderate	28 days after single dose	Participant lost to follow-up
2	60 mcg<br>18-50 years	Neutropenia	Mild	33 days after first dose	51 days
3	60 mcg<br>55-70 years	Lymphopenia	Mild	28 days after first dose	56 days
4	60 mcg<br>55-70 years	Aspartate aminotransferase increased	Mild	28 days after second dose	14 days
5	60 mcg<br>55-70 years	Leukopenia	Mild	28 days after first dose	56 days

* All AEs related to H10ssF vaccination resolved by the next study visit. Participant 1 did not have an additional hematological evaluation before being lost to follow-up.

Supplementary Figures

Supplementary Figure 1: H10ssF vaccination did not elicit antibody responses against human ferritin antigens.

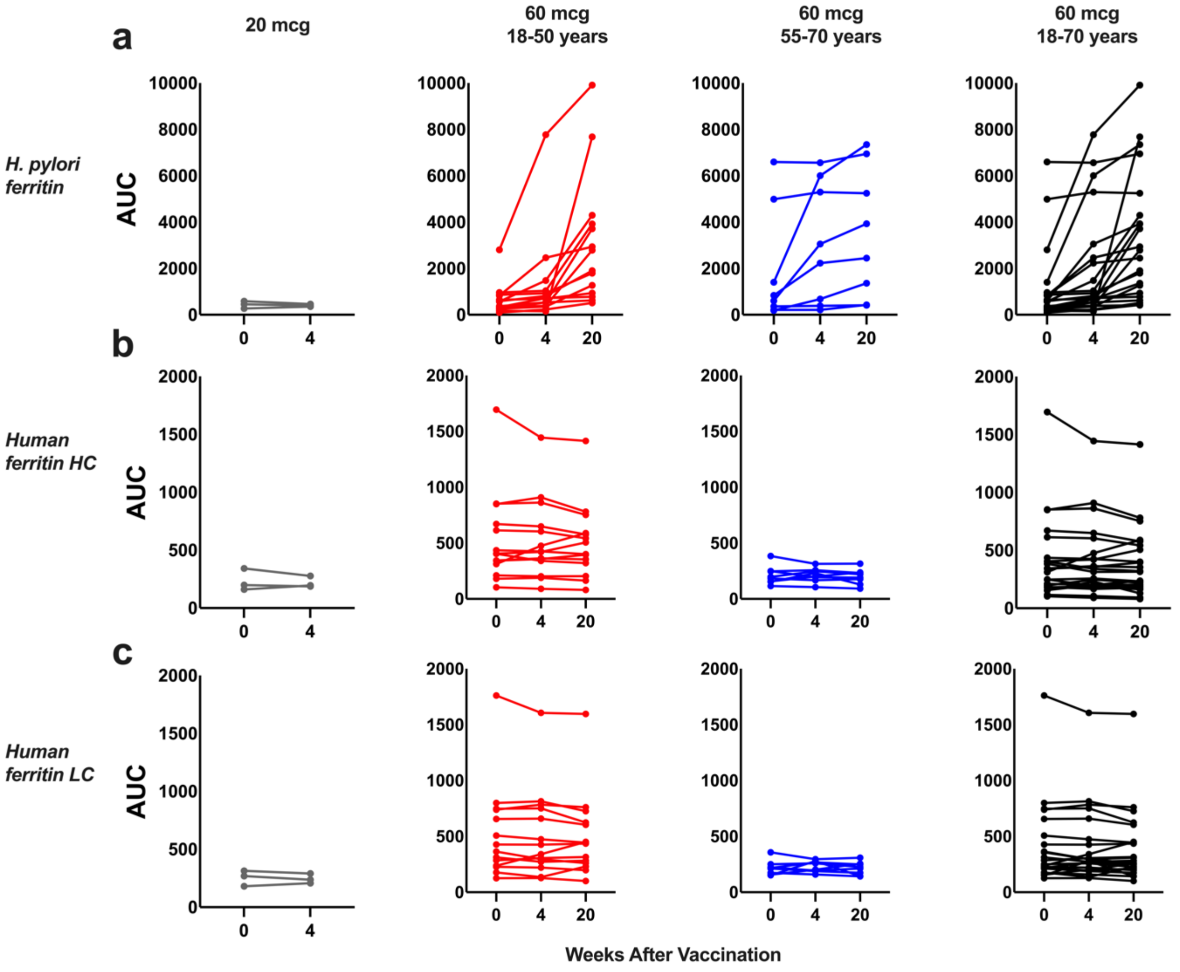

Antibodies binding (a) *Helicobacter* (*H.*) *pylori* non-heme ferritin or the (b) human ferritin heavy chain (HC) or (c) human ferritin light chain (LC) proteins were assessed by ECLIA. Participants are stratified in columns by vaccine dose and age range, with the far-right column including all participants who received two doses of 60 mcg H10ssF. AUC: area under the curve.

Supplementary Figure 2: Binding and neutralizing antibody responses following a single 20 mcg dose of H10ssF.

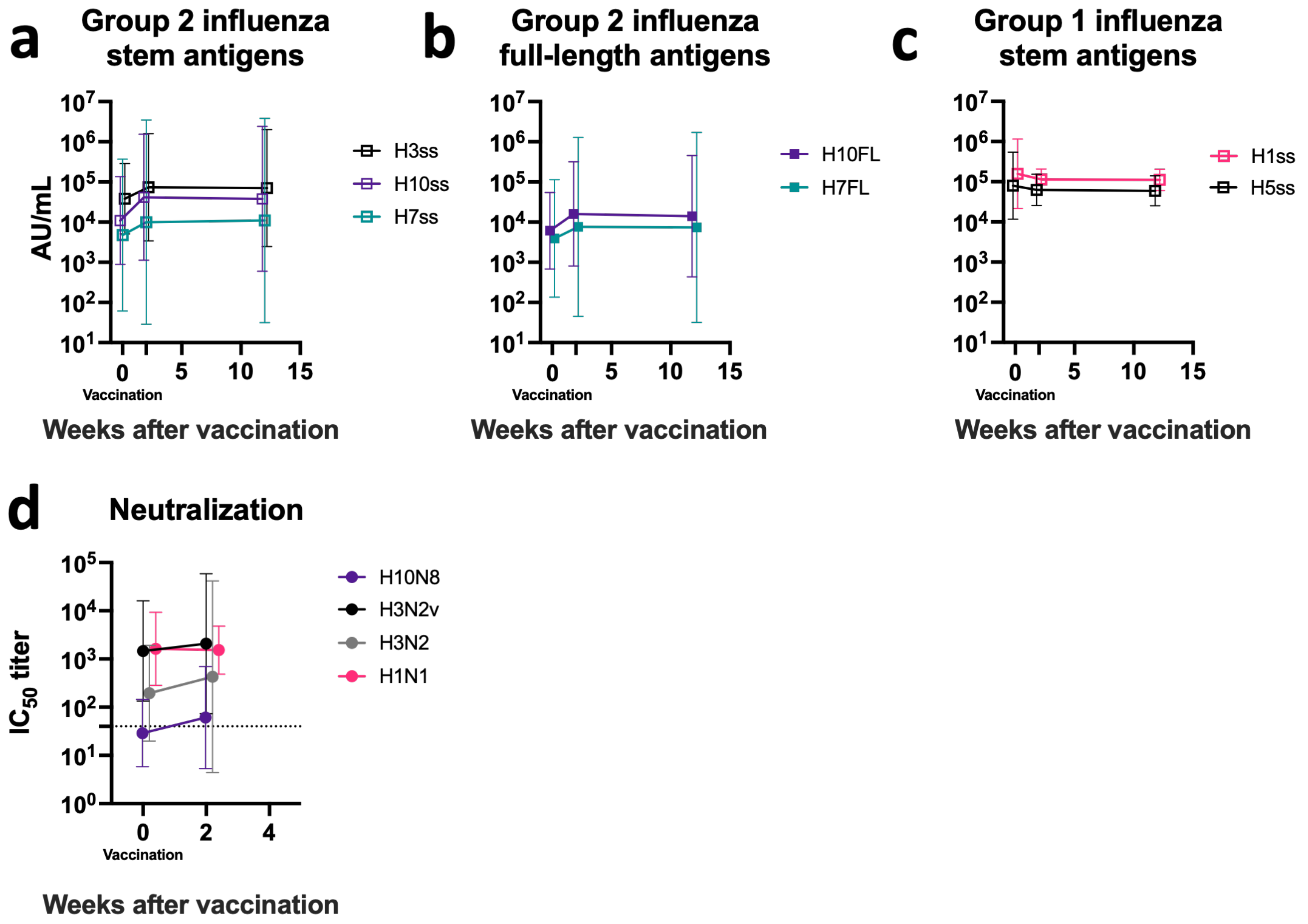

Binding antibodies were assayed by ECLIA using group 2 influenza stabilized stem antigens (a), group 2 influenza full-length antigens (b), or group 1 influenza stabilized stem antigens (c). The GM AU/mL of the 20 mcg recipients' (*n*=3) binding antibodies are shown along with 95% CI. Neutralizing antibodies were assessed by microneutralization assay (d). The GMT of the 20 mcg recipients' (*n*=3) neutralizing antibodies are shown along with 95% CI. Dotted line indicates the limit of detection (LOD). No significant differences from baseline were detected by ECLIA or microneutralization assay at any time after vaccination.

Supplementary Figure 3: H10ssF vaccination elicited neutralizing antibodies against H10N8 A/Jiangxi-Donghu/346/2013 influenza virus.

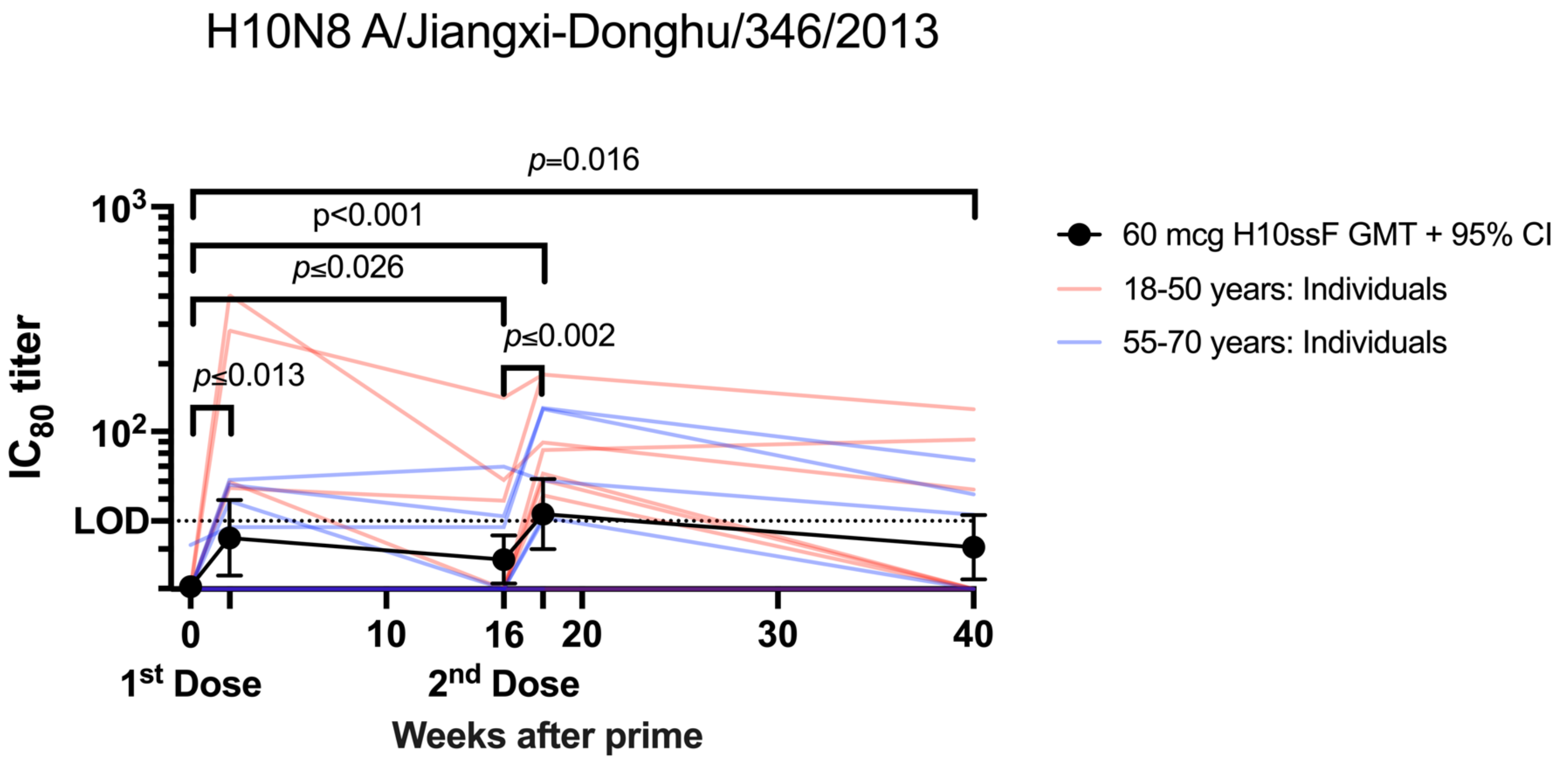

The geometric mean IC_{80} neutralizing antibody titers (GMT) and 95% CI of all 60 mcg H10ssF recipients are shown over time (*x* axis) in black, with individual participants' titers in red or blue based on their age group. Dotted line indicates the LOD. Brackets indicate a significant difference between the indicated timepoints, with the *p* value noted above the line. Where LOD imputation with zero produced a different *p* value than imputation with half LOD, results are reported as $p \leq x$, where x is the least significant *p* value resulting from the two test methods.

Supplementary Figure 4: H10ssF vaccination elicited H10-specific antibodies with Fc-mediated activity.

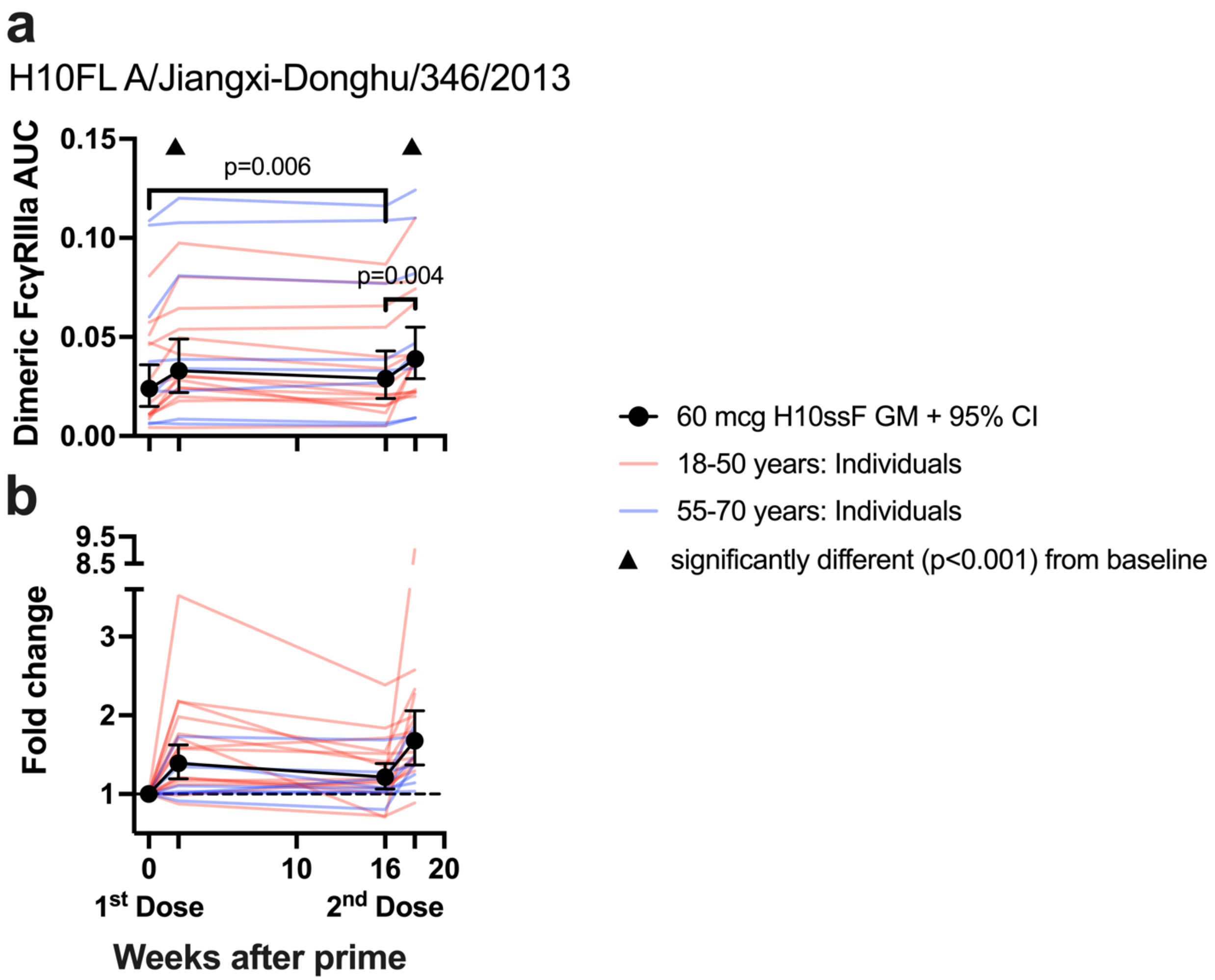

The results of a dimeric FcγRIIIa binding assay are shown (a) in black by geometric mean and 95% CI of the area under the curve (AUC) of serial dilutions of serum with individual participants' values in red or blue based on age group. Brackets indicate a significant difference between the indicated timepoints, with the *p* value noted above the line. Where the significance between a timepoint and baseline is $p<0.001$, this is noted by a symbol above the later timepoint. Fold changes of the AUC are below, in (b).

Supplementary Figure 5: H3N2-neutralizing antibody response to H10ssF vaccination

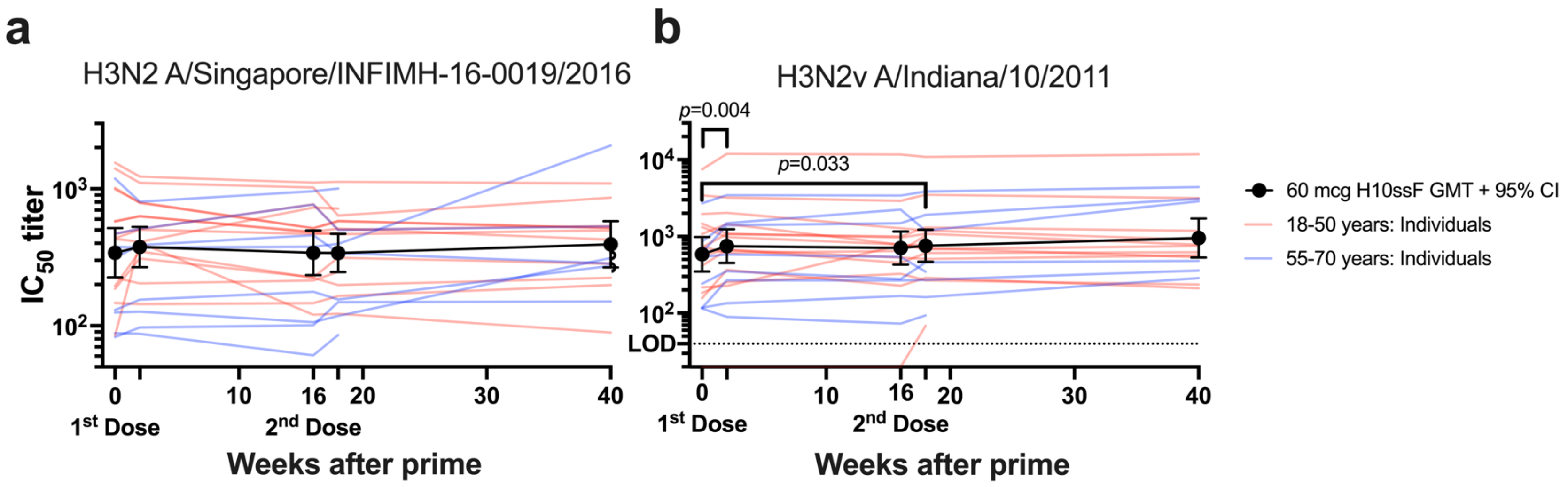

(a) H3N2 A/Singapore/INFIMH-16-0019/2016 and (b) H3N2v A/Indiana/10/2011 IC_{50} neutralizing antibody GMT and 95% CI are shown in black over time (*x* axis), with individual participants' values in red or blue based on age group. In (b), the dotted line indicates the LOD. Brackets indicate significant results of paired t tests between two time points with the *p* value noted above the line.

Supplementary Figure 6: Significant increases in geometric mean binding antibody responses were not observed against group 1 influenza HAs following H10ssF vaccination.

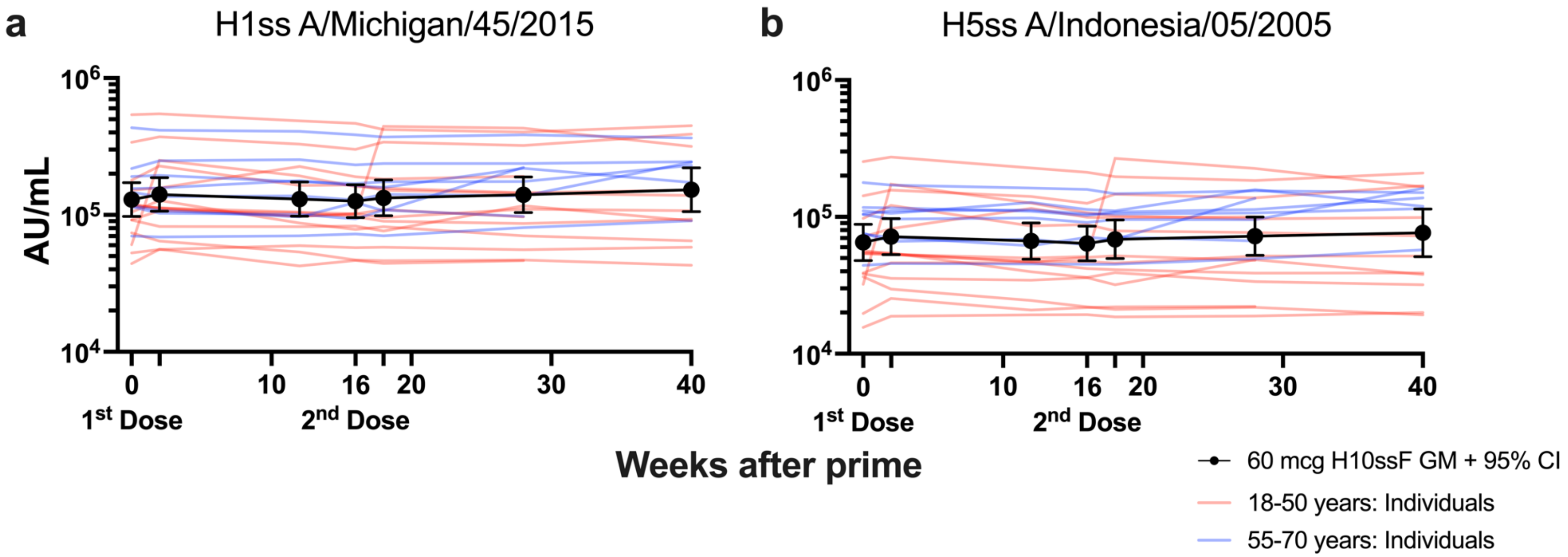

Binding antibodies were assayed using the H1 stabilized stem (H1ss, a) or H5ss (b) antigens by ECLIA. The GM of the 60 mcg recipients' binding antibodies in AU/mL are shown with 95% CI in black, with individual participants' concentrations in red or blue based on their age group.

Supplementary Figure 7: H10ssF vaccination did not elicit neutralizing antibodies against H1N1 A/Michigan/45/2015.

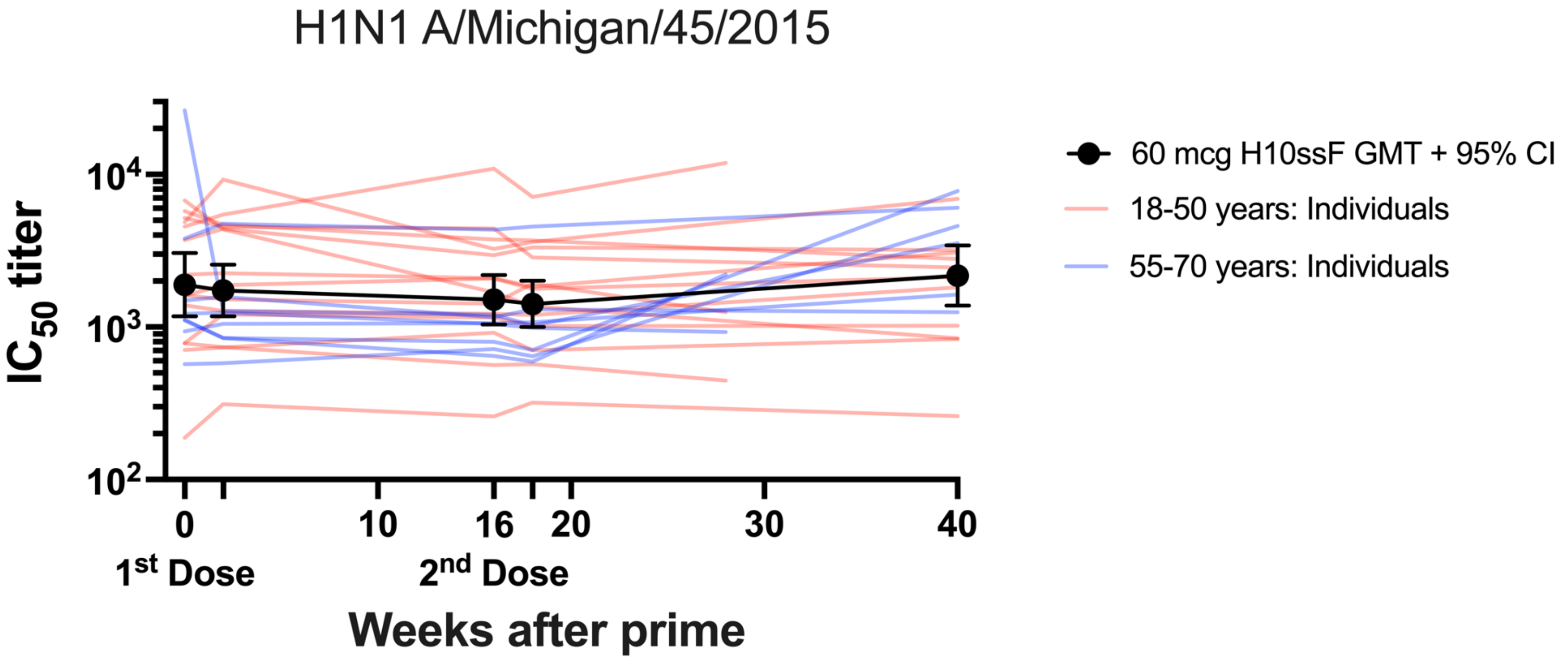

IC_{50} neutralizing antibody GMT and 95% CI are shown in black over time (*x* axis), with individual participants' values in red or blue based on age group.

Supplementary Figure 8: H10ssF vaccination elicited H7 cross-reactive antibodies with Fc-mediated activity.

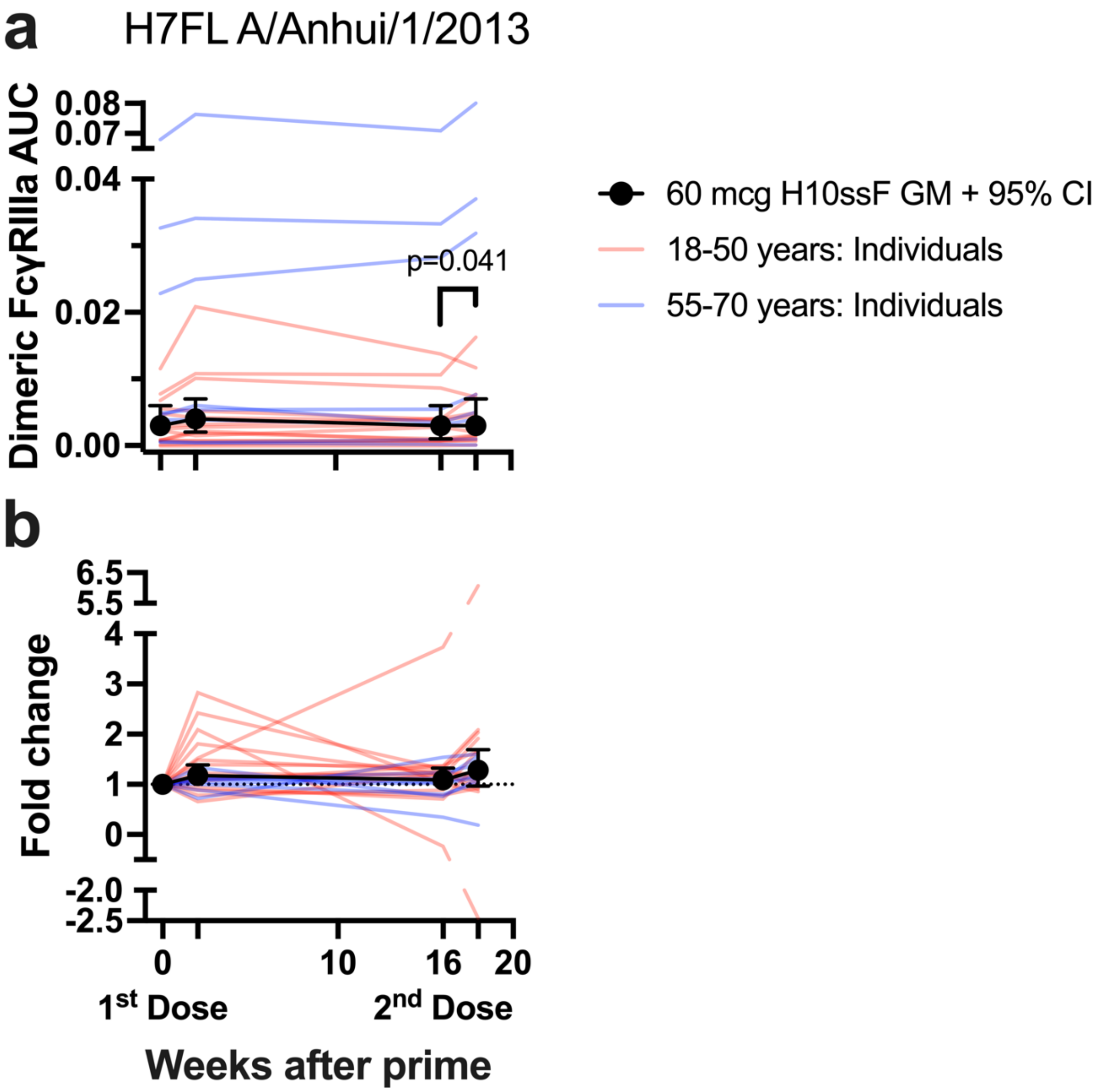

The results of a dimeric FcγRIIIa binding assay are shown (a) in black for H7FL by GM and 95% CI of the area under the curve (AUC) of serial dilutions of serum with individual participants' values in red or blue based on age group. Brackets indicate two time points that are significantly different, with the *p* value noted above the line. Fold changes of the AUC are below (b). In (b) the dashed line represents no fold change over baseline.

Supplementary Figure 9: Participant age had little to no impact on antibody responses for all antigens tested.

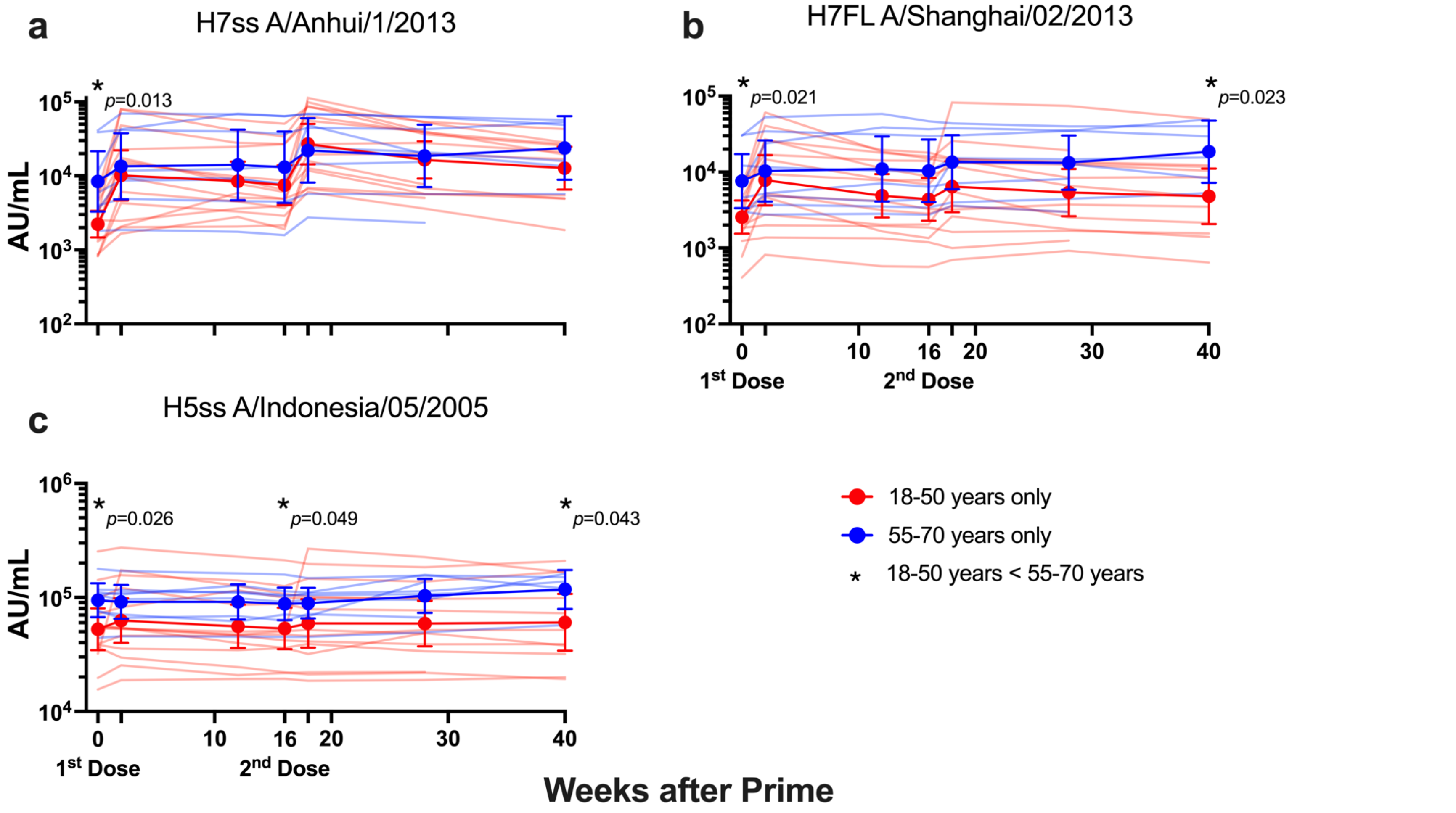

Binding antibodies were assayed using the H7ss (a) H7FL (b) or H5ss (c) antigens by ECLIA. The GM of the 60 mcg recipients' binding antibodies in AU/mL are shown with 95% CI with thick lines in red for 18-50 year old participants, and in blue for 55-70 year old participants, with individual participants' concentrations shown by thin red or blue lines, respectively. Statistical differences between the two age groups are noted by black stars over the sampling time of the difference.
